# Refractory CMV Enteritis in Small Bowel Transplantation: A Case Highlighting the Challenges of Balancing Immunosuppression and Novel Antiviral Therapies

**DOI:** 10.3390/v17101379

**Published:** 2025-10-15

**Authors:** Abdulrahman A. Al-Saud, Ehab H. Abufarhaneh, Madain S. Alsanea, Reem M. Alameer, Amani H. Yamani, Fatimah S. Alhamlan, Reem S. Almaghrabi

**Affiliations:** 1 Organ Transplant Centre of Excellence, King Faisal Specialist Hospital and Research Centre, P.O. Box 3354, Riyadh 11211, Saudi Arabia; ehab@kfshrc.edu.sa (E.H.A.); f1504050@kfshrc.edu.sa (R.M.A.); ayamani1@kfshrc.edu.sa (A.H.Y.); ramaghrabi@kfshrc.edu.sa (R.S.A.); 2 Research & Innovation Department, King Faisal Specialist Hospital and Research Centre, Riyadh 11211, Saudi Arabia; maalsanea@kfshrc.edu.sa (M.S.A.); falhamlan@kfshrc.edu.sa (F.S.A.)

**Keywords:** small bowel transplant, cytomegalovirus, CMV enteritis, refractory CMV, maribavir

## Abstract

**Background:** Cytomegalovirus (CMV) remains a formidable complication in small bowel transplantation (SBT) due to the graft’s high immunogenicity and profound immunosuppression required, with refractory disease representing a particularly devastating challenge. **Case:** We report an 18-year-old male who underwent SBT, complicated by recurrent acute rejection episodes requiring intensive immunosuppression. He developed refractory CMV disease, marked by non-response to first line therapy with ganciclovir—despite the absence of genotypic resistance—necessitating sequential use of foscarnet, dual antivirals, CMV immunoglobulin, and novel agents (maribavir and letermovir). **Discussion:** This case illustrates the multifactorial drivers of refractory CMV disease in SBT recipients, including donor–recipient serostatus mismatch, profound immunosuppression through T-cell-depleting induction, corticosteroid exposure, and biologic therapy. It highlights the distinction between refractory and resistant CMV, and the role of combination antiviral strategies including novel agents to achieve disease control. Outcomes remain dismal despite aggressive and innovative therapies, underscoring the limited efficacy of interventions in the context of severe immunologic compromise. **Conclusions:** Refractory CMV enteritis in SBT exemplifies the extreme difficulty of balancing viral control with rejection management. Despite exhausting antiviral strategies, survival remains poor. **Highlights:** Refractory CMV enteritis is a significant challenge in small bowel transplant recipients due to intense immunosuppression. Persistent CMV disease may occur despite antiviral prophylaxis and the absence of resistant gene mutations. Combination antiviral strategies, including maribavir, demonstrated significant clinical improvement. Profound immunosuppression required to manage acute graft rejection episodes complicates antiviral management and disease clearance. Despite best efforts in CMV management in this population, outcomes may still be compromised by unrelated or compounding factors.

## 1. Introduction

Cytomegalovirus (CMV) remains one of the most clinically significant viral pathogens in the setting of solid organ transplantation (SOT) [1], with an estimated global seroprevalence of 60–90% in adults [2,3]. In immunocompetent hosts, infection is often self-limiting; however, in immunocompromised individuals, CMV is associated with a wide spectrum of clinical manifestations ranging from asymptomatic viremia to life-threatening end-organ disease [4].

The immune response to CMV is orchestrated by innate and adaptive cellular mechanisms. The virus establishes latency within myeloid lineage cells, and its reactivation is tightly controlled by robust T cell-mediated immunity. Control of viral dissemination relies on virus-specific CD8^+^ cytotoxic T lymphocytes, which directly eliminate infected cells, and CD4^+^ T helper cells, which sustain cytotoxic responses and promote antiviral antibody production [5]. In the setting of SOT, this balance is disrupted by T-cell-depleting therapies [6]. Consequently, viral reactivation proceeds unchecked, leading to direct cytopathic tissue injury and to indirect immunomodulatory effects increasing the risk of acute and chronic allograft rejection, susceptibility to opportunistic infections, and impairment of overall survival [1].

Among all forms of SOT, small bowel transplantation (SBT) recipients remain uniquely vulnerable to CMV infection, with reported incidence rates of up to 40% [7], and enteritis representing one of the most severe presentations [8]. The intestinal graft is highly immunogenic, enriched with gut-associated lymphoid tissue and densely populated with mucosal immune cells, making it both prone to rejection requiring intense immunosuppression and an ideal reservoir for viral replication [9,10,11].

The therapeutic landscape for CMV has historically relied on ganciclovir or valganciclovir as first-line therapy. Alternative antivirals, such as foscarnet and cidofovir, provide options in cases of resistance but are limited by adverse effects [1]. More recently, the advent of novel agents—such as maribavir for the treatment of refractory or resistant disease and letermovir for prophylaxis—has expanded the management strategies [1].

Despite these advances, CMV disease remains a formidable obstacle associated with high mortality [4]. A recent study found that patients with CMV viremia occurring within 6–12 months post-intestinal transplant had 0% survival at three years [12].

The case presented here underscores these realities, and contributes to the very limited but growing body of literature on refractory CMV enteritis in intestinal transplantation.

## 2. Clinical Case

**Figure 1:**
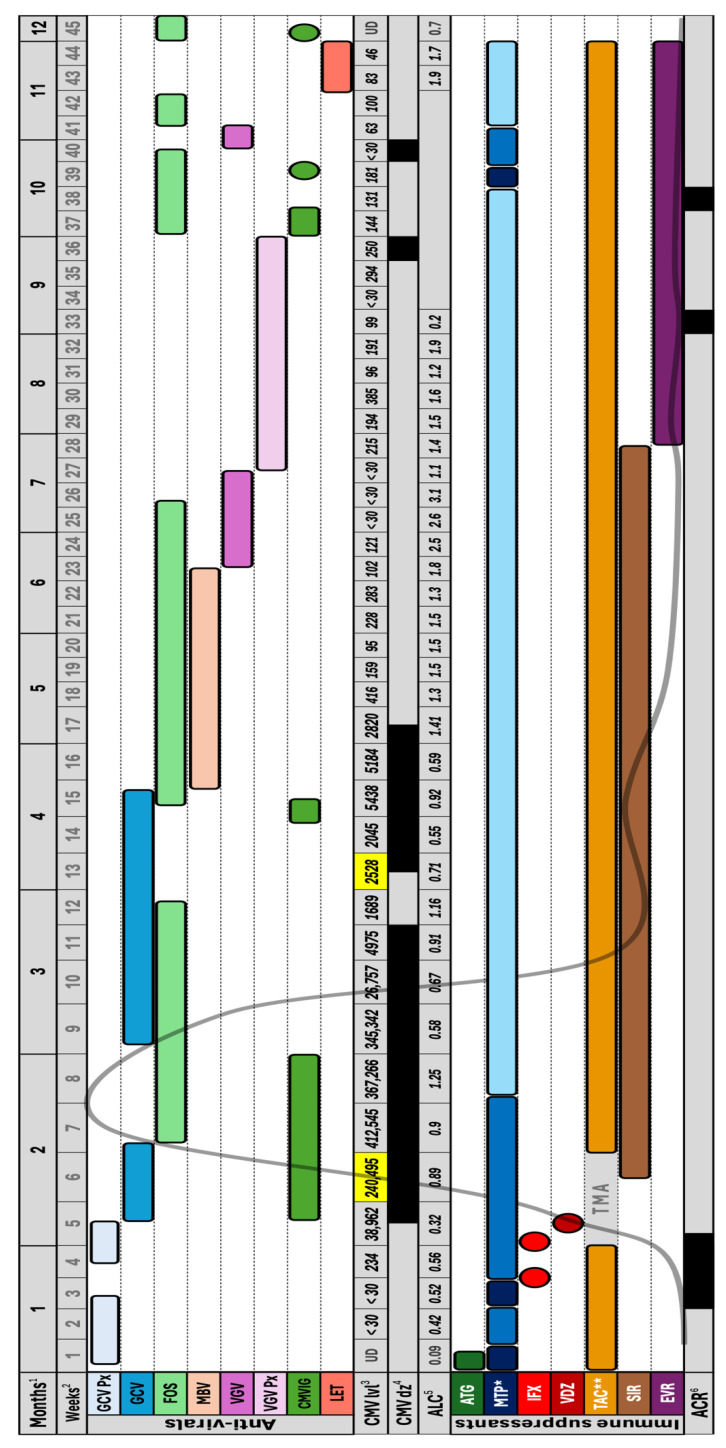
Abbreviations: GCV Px: Ganciclovir prophylaxis (5 mg/kg daily), GCV: Ganciclovir (5 mg/kg BID), FOS: Foscarnet (60 mg/kg TID), MBV: Maribavir (400 mg BID), VGV: Valganciclovir (900 mg BID), VGV Px: Valganciclovir prophylaxis (900 mg daily), CMVIG: Cytomegalovirus immunoglobulin, LET: Letermovir (480 mg daily), CMV lvl: Cytomegalovirus DNA level, UD: Undetectable, CMV dz: Cytomegalovirus disease (enteritis), ALC: Absolute lymphocyte count, ATG: Anti-thymocyte globulin, MTP: Methylprednisolone, IFX: Infliximab, VDZ: Vedolizumab, TAC: Tacrolimus, TMA: Thrombotic microangiopathy, SIR: Sirolimus, EVR: Everolimus, ACR: Acute cellular rejection. 1: Months post-transplant. 2: Weeks post-transplant. 3: Weekly blood CMV DNA level in international units/mL. Highlighted in yellow is when CMV resistance genotyping was performed (weeks 6 & 13), and showed no resistance mutations. 4: CMV disease (enteritis) presence highlighted in black, based on histopathology from surveillance endoscopies performed every week or so throughout the patient’s course. 5: Absolute lymphocyte count in 10^9^/L. It was not obtained in the period between 34 and 42 weeks post-transplant. 6: Acute cellular rejection presence highlighted in black based on pathology from surveillance endoscopic biopsies performed every week or so throughout the patient’s course. * Methylprednisolone pulse (125–500 mg) in dark blue, moderate-high dose (0.5–1 mg/kg) in blue, and maintenance dose (~0.2 mg/kg) in light blue. ** Tacrolimus was held for 2 weeks (weeks 6 & 7) due to thrombotic microangiopathy. The curved grey line throughout the figure reflects the rise and fall in CMV DNA level.

Our patient was an 18-year-old male with a history of short bowel syndrome following multiple resections for small bowel volvulus. He underwent SBT in 2023, which included ileostomy formation. His weight was 35 kilograms (kg). The donor was CMV IgG seropositive, while the recipient’s pre-transplant CMV IgG was equivocal. Induction immunosuppression consisted of methylprednisolone 500 milligrams (mg) once, followed by gradual tapering, and rabbit antithymocyte globulin (ATG) 1.5 mg/kg for three days, followed by tacrolimus maintenance [Figure 1]. Tacrolimus trough concentrations remained within the therapeutic range throughout his course. In the immediate postoperative period, he was started on ganciclovir prophylaxis (5 mg/kg daily); however, prophylaxis was withheld for a few days due to laboratory abnormalities suggestive of acute kidney injury. It was subsequently re-initiated following confirmation of normal creatinine clearance by cystatin-C testing.

**Figure 2:**
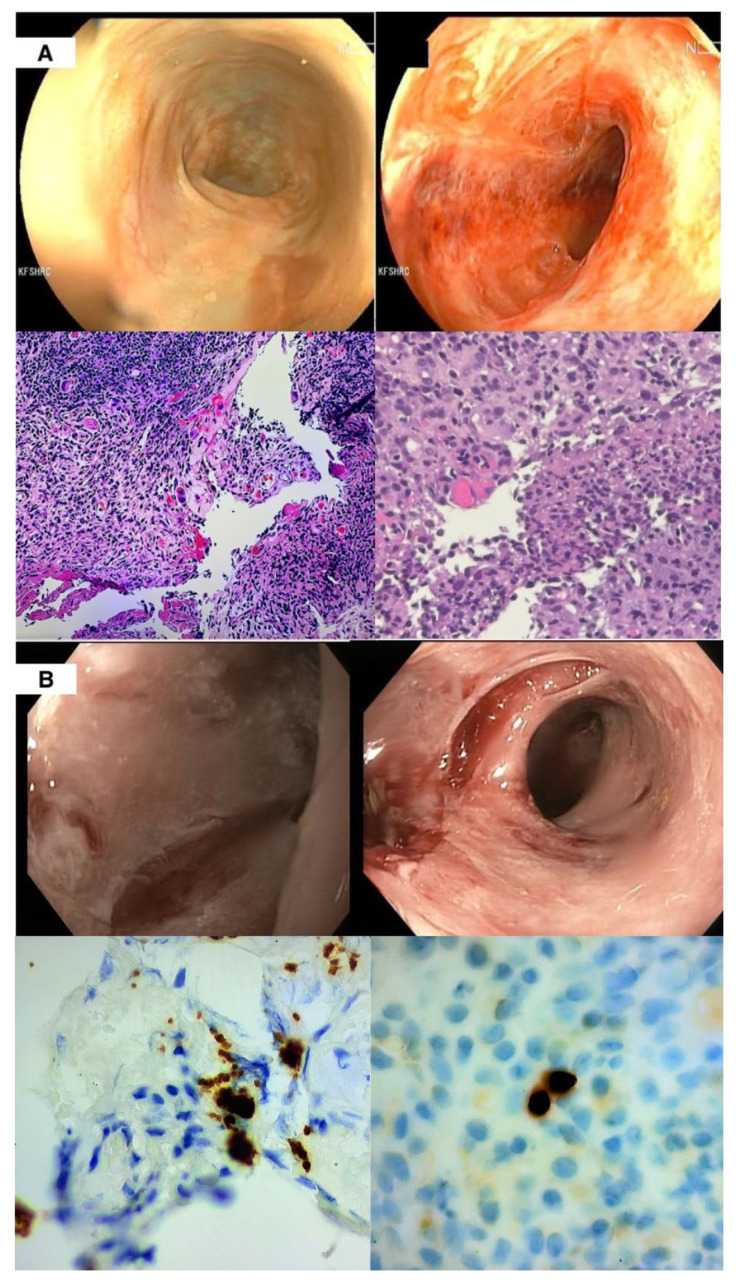
(**A**) Top: two images taken during endoscopy of the patient’s transplanted small bowel showing severe ulceration, granulation tissue and inflammatory exudate. Bottom: two images from the biopsy histopathology showing diffuse mucosal injury with marked lymphocytic infiltration and crypt epithelial apoptosis consistent with acute rejection. Immunohistochemistry (IHC) staining was negative for CMV. (**B**) Top: two images taken during endoscopy of the patient’s transplanted small bowel showed an inflamed graft with extensive ulceration at the anastomosis. Bottom: two images from the biopsy showing IHC staining positive for CMV.

The early post-transplant course was marked by significant complications. He experienced an episode of acute cellular rejection (ACR) [Figure 2A], diagnosed by surveillance endoscopies through the stoma performed at weekly intervals. Management consisted of five days of methylprednisolone (500 mg daily), followed by two doses of infliximab (5 mg/kg each) for persistent ACR, and a single dose of vedolizumab (300 mg). He also developed thrombotic microangiopathy (TMA), presumed secondary to tacrolimus, necessitating multiple sessions of plasma exchange with fresh frozen plasma, while tacrolimus was withheld for two weeks. In the context of limited therapeutic alternatives, an mTOR inhibitor (sirolimus) was initiated during recovery from TMA, prior to resumption of tacrolimus.

The patient was monitored for CMV reactivation on a weekly basis using CMV DNA polymerase chain reaction (PCR). Between the ACR and TMA events, surveillance revealed an increasing serum CMV DNA level (234 International units/mL) on day 25 post-transplant. He remained on ganciclovir prophylaxis, but in the following week CMV DNAemia significantly worsened, along with endoscopic biopsies demonstrating numerous CMV inclusions [Figure 2B]. This prompted escalation from prophylaxis to induction-dose intravenous ganciclovir (5 mg/kg twice daily) alongside CMV immunoglobulin (CMVIG). Immunoglobulin levels were not available at that time. Despite nearly two weeks of therapy, viremia and tissue disease persisted, prompting a switch to intravenous foscarnet due to suspected ganciclovir resistance.

Resistance testing was performed via PCR and Sanger sequencing (3730xl DNA Analyzer, Applied Biosystems, Foster city, CA, USA), targeting the UL97, UL54, and UL56 genes. Sequencing demonstrated high-quality reads but no resistance mutations. Given the suboptimal response to foscarnet monotherapy, ganciclovir was re-introduced in combination with foscarnet. This dual regimen led to marked clinical and virological improvement and was maintained for three weeks, before foscarnet was discontinued.

CMV disease relapsed in the fourth month post-transplant despite stable immunosuppression and appropriate ganciclovir dosing, necessitating resumption of dual therapy. Serum IgG level was within normal range (11.6 g/L). Repeated CMV resistance testing was still detecting no mutations. At this point, maribavir was requested and replaced ganciclovir in combination with foscarnet. This regimen produced a remarkable clinical response, with resolution of CMV enteritis. Following completion of the maribavir course, he was transitioned to valganciclovir while continuing foscarnet until CMV DNA level became undetectable. Subsequently, he remained on valganciclovir monotherapy, later reduced to prophylactic dosing for maintenance.

At nine months post-transplant, the patient experienced another episode of acute graft rejection. Due to concurrent infected intra-abdominal collections requiring surgical evacuation, augmentation of immunosuppression was deferred. At that point, sirolimus had been substituted by everolimus, owing to its potentially more favorable wound-healing profile, with the dose increased incrementally from 0.5 mg twice daily to 4 mg twice daily. Two weeks later, he developed a recurrence of CMV enteritis. As ganciclovir was not available at that time, he was treated with foscarnet in combination with CMVIG, achieving subsequent improvement. One more episode of acute graft rejection occurred at ten months post-transplant, treated this time with methylprednisolone for three days (250 mg daily, followed by gradual tapering). Subsequently, a relapse in CMV enteritis occurred requiring a repeated course of foscarnet for management.

After disease control, foscarnet was discontinued and the patient was transitioned to letermovir prophylaxis. Despite sustained virological suppression, he developed bowel perforation and septic shock one month later, necessitating explantation of a non-viable transplanted graft. Unfortunately, he did not survive this complication.

## 3. Discussion

CMV infection continues to pose one of the greatest challenges in small bowel transplantation, where the interplay between viral replication, host immunity, and immunosuppressive therapy creates a precarious balance [1,9]. In this patient, several factors contributed at different stages to the onset and progression of CMV disease, each influencing the overall clinical course in distinct ways. In the following discussion, we explore these factors and their potential contribution to the patient’s course.

### 3.1. Host Risk Factors and Induction Immunosuppression

The pathogenesis of CMV disease in our patient was likely multi-factorial. To start, in addition to the inherent high risk of CMV reactivation in small bowel transplantation, he presented with an equivocal CMV serostatus while receiving a graft from a seropositive donor; under contemporary guidance, equivocal recipient serostatus is managed as seronegative, thereby assigning the higher-risk category and increasing the likelihood of primary infection and severe end-organ disease [1,11,13]. This was further compounded by induction with ATG, which depletes T-cell populations crucial for viral immune surveillance, and by the subsequent need for intensive treatment of acute rejection with corticosteroids, further diminishing CMV-specific T-cell responses and setting the stage for uncontrolled viral replication. The patient also received biologic therapy for acute rejection, specifically infliximab and vedolizumab, but in contrast to the well-established independent association of ATG and corticosteroids with CMV reactivation in the literature [14,15], evidence regarding biologic agents remains inconclusive.

### 3.2. Biologic Therapy and the Risk of CMV Reactivation

Biologic agents have been used in SBT recipients as an adjunctive therapy for cases of refractory ACR [16,17,18]. Infliximab is an anti-tumor necrosis factor (TNF) monoclonal antibody that binds and neutralizes soluble and membrane TNF-α, preventing TNF receptors I and II activation and downstream pro-inflammatory signaling [19]. Evidence on the effect of infliximab on CMV reactivation is largely derived from cohorts of patients with inflammatory bowel disease (IBD), in which no significant risk has been demonstrated. For instance, a 2021 meta-analysis of 20 studies in patients with ulcerative colitis (UC) found no statistically significant association between infliximab and CMV reactivation (pooled OR, 1.92; 95% CI, 0.87–4.22), whereas other therapies—particularly systemic corticosteroids—were associated with higher risk [20].

In contrast, studies specifically evaluating this effect in SOT recipients are limited. A 2021 systematic review of case reports and series on the use of biologic therapy in SOT recipients with chronic inflammatory diseases found that CMV infection or reactivation was the most frequent viral complication, occurring in 7 of 88 liver or renal transplant recipients (7.9%), predominantly in those receiving infliximab [21].

With respect to CMV reactivation in SBT recipients treated with infliximab, available data remain scarce. Avsar et al. reported a case of CMV enteritis in a SBT recipient with refractory rejection who received infliximab; CMV did not worsen during therapy, but rejection persisted, ultimately necessitating graft explantation despite virologic recovery [22].

Vedolizumab, on the other hand, is an anti-integrin monoclonal antibody that acts selectively in the gut, specifically targeting α4β7 integrin expressed by a subset of gastrointestinal-homing T lymphocytes [23].

Data extrapolated from patients with IBD suggest a higher risk of CMV reactivation with vedolizumab compared to infliximab. For instance, a single-center study of 33 CMV-seropositive patients with UC treated with either infliximab or vedolizumab evaluated the risk of CMV reactivation and demonstrated a higher incidence among those receiving vedolizumab compared with infliximab [24]. In addition, Meeralam et al. reported an unusual case of CMV colitis in a patient with UC receiving vedolizumab monotherapy [25], raising the possibility of an isolated role of vedolizumab as a risk factor for CMV.

Evidence on CMV reactivation with vedolizumab in SOT recipients is also limited. A recent meta-analysis in SOT recipients with IBD reported higher rates of infectious complications with vedolizumab compared to infliximab, including CMV [26], although small bowel transplant recipients were not represented.

In conclusion, the evidence regarding biologic therapy and the risk of CMV reactivation remains conflicting, particularly as most available data are derived from IBD populations, which differ substantially from SOT recipients in both the context of immunosuppression and the pathophysiology of CMV infection. Our aim is to underscore the complexity of immunosuppressive management in SBT. Importantly, based on this single case, no causal association between biologic therapy and CMV reactivation can be established, as the worsening of CMV disease may reflect the profound overall immunosuppression, though the effect warrants further study.

### 3.3. The Effect of Maintenance Immunosuppression on CMV Reactivation

Tacrolimus, a calcineurin inhibitor, is a cornerstone of maintenance immunosuppression across SOT [27]. With respect to CMV, contemporary evidence indicates that risk is driven primarily by host factors and overall intensity of immunosuppression rather than tacrolimus as an isolated factor [1]. Nevertheless, higher tacrolimus exposure has been associated with increased CMV events in some cohorts—including an intestinal transplant series in which greater average tacrolimus levels independently predicted the first episode of CMV disease [8]—supporting the view that excess immunosuppression, rather than the agent itself, underlies risk. This consideration was not pertinent in our patient, as tacrolimus trough concentrations remained within the therapeutic range throughout his course.

mTOR inhibitors (sirolimus, everolimus) form a complex with FKBP12 that inhibits mTORC1, thereby arresting cell-cycle progression and blunting T-cell proliferation [28]; they are widely used as maintenance agents in SOT. Accumulating clinical evidence indicates lower CMV DNAemia and disease with mTOR-inhibitor-based regimens versus mycophenolate/CNI regimens [29], consistent with CMV’s reliance on host mTOR signaling. A meta-analysis assessing CMV risk across mTOR inhibitors demonstrated robust anti-CMV efficacy overall and suggested greater protection with everolimus than with sirolimus [30]. Current CMV guidelines even suggest conversion to an mTOR inhibitor to reduce recurrent CMV in selected kidney recipients [1]. Data specific to SBT are sparse: case series document substantial CMV burden overall but do not identify mTOR inhibitors as an isolated risk factor [9]; only isolated reports describe CMV events on everolimus without clear attribution [31]. Overall, mTOR inhibitors are not associated with increased CMV risk and may be protective, though SBT-specific data remain limited. In our patient, we did not observe any clear worsening of CMV disease attributable to mTOR inhibitor therapy.

### 3.4. CMV Prophlyaxis and the Onset of Infection

The epidemiology of CMV disease in transplantation has evolved in recent decades with the widespread adoption of antiviral prophylaxis [1]. Universal prophylaxis with valganciclovir or intravenous ganciclovir has significantly reduced the incidence of CMV disease in SOT populations overall. In this case, a transient lapse in antiviral prophylaxis may have been an immediate trigger for CMV replication, underscoring that even brief interruptions can precipitate significant clinical disease in this particularly high-risk population. However, regardless of prophylaxis, breakthrough infections have been reported and remain a persistent problem in high-risk subgroups such as donor-seropositive/recipient-seronegative (D+/R−) or sero-equivocal recipients, and SBT recipients. For example, Fernandez et al. noted in their cohort of SBT recipients an incidence of 20% of breakthrough CMV infection despite ganciclovir or valganciclovir prophylaxis [13], reaffirming the disproportionate risk and vulnerability to CMV disease in this population.

### 3.5. Refractory CMV Disease

The patient’s persistent viremia despite appropriately dosed ganciclovir raised the suspicion of antiviral resistance, leading to foscarnet initiation. However, resistance testing revealed no UL97, UL54 or UL56 gene mutations, highlighting the distinction between resistant and refractory CMV disease [32]. Resistant CMV is driven by viral genetic mutations that confer reduced susceptibility, while refractory CMV refers to clinical non-response despite appropriate therapy for two weeks or more, often due to high viral burden or profound host immune deficiency [1]. In this case, the absence of resistance mutations combined with poor virological clearance suggested refractory disease, a phenomenon increasingly recognized in transplant recipients. Functional antiviral failure of this nature has been documented in up to 10% of infected SOT recipients [33], though its incidence in intestinal transplantation specifically remains undefined.

The management of refractory CMV disease is particularly challenging in SBT where viral burden is often higher, mucosal tissue involvement more severe, and immunosuppression more profound [4]. The dual regimen of ganciclovir and foscarnet used in this case was associated with marked virological improvement, consistent with prior observations of synergistic activity between the two agents [1,32]. Nevertheless, this combination is limited by adverse effects including myelosuppression, nephrotoxicity and electrolyte imbalance [1], and its prolonged use in SBT recipients who often have concomitant renal impairment is not sustainable. Adjunctive CMVIG was also administered, a strategy that may transiently augment viral clearance by enhancing antibody-mediated immunity, though data supporting its efficacy remain inconsistent [1]. Evidence supporting CMVIG in refractory or resistant CMV after SOT is low in quality; it may be used as an adjunct in severe tissue-invasive disease, as in our patient, or hypogammaglobulinemia but is not recommended as routine therapy [1,34].

### 3.6. Novel Antiviral Therapy

Maribavir, a novel oral UL97 kinase inhibitor with a distinct mechanism of action compared to traditional antivirals, interferes with viral DNA synthesis, encapsidation, and nuclear egress [35]. Maribavir has been shown in randomized clinical trials to be superior to investigator-assigned therapy for refractory or resistant CMV, with fewer hematologic and renal toxicities [36]. Our patient also achieved marked clinical and virological improvement once maribavir was introduced in combination with foscarnet, underscoring its potential utility in severe, tissue-invasive disease. Liu et al. were the first to report the successful and safe management of CMV in an intestinal transplant recipient with ganciclovir-refractory infection using maribavir monotherapy [37]. Notably, genotypic resistance testing was not performed in that case. While maribavir is generally recommended as monotherapy [36], in our case, combination therapy was favored due to the high viral burden and severe end-organ involvement. This decision reflects a pragmatic approach that aligns with evolving practices in complex transplant cases, where combination regimens are increasingly considered to maximize viral suppression while limiting resistance emergence and toxicities [1]. To our knowledge, this is the first reported SBT recipient case in which maribavir was utilized as combination therapy for refractory CMV disease.

Letermovir, an inhibitor of the CMV terminase complex, was later introduced as prophylaxis once virological control was achieved. Although its use in SBT remains off-label, growing evidence suggests it may serve as an effective maintenance therapy in high-risk solid organ transplant recipients [38]. This strategy may be particularly relevant in patients with prior refractory disease, where the risk of relapse is substantial [33]. In our case, letermovir provided a period of virological stability before the patient ultimately succumbed to non-viral complications.

## 4. Conclusions

Refractory CMV enteritis after small bowel transplantation remains a devastating complication; despite successfully managing CMV through the use of combination, adjunctive, and novel antiviral therapies, outcomes may remain poor in these profoundly immunosuppressed patients. This case illustrates the complexity of managing such high-risk individuals and adds to the limited literature in this population.

## Data Availability

The original contributions presented in this study are included in the article. Further inquiries can be directed to the corresponding author(s).
